# Long-Term Efficacy of Single-Incision Laparoscopic Intragastric Resection in Treating Gastrointestinal Stromal Tumors

**DOI:** 10.5152/tjg.2024.24080

**Published:** 2024-03-01

**Authors:** Veysel Umman, Özgür Kılınçarslan, Recep Temel, Yener Bağdat, Sarp Tunalı, Sinan Ersin, Taylan Özgür Sezer, Özgür Fırat

**Affiliations:** Department of General Surgery, Ege University, İzmir, Turkey

**Keywords:** Laparoscopic, intragastric resection, gastrointestinal stromal tumor, gist

## Abstract

**Background/Aims:**

Gastrointestinal stromal tumors (GISTs), the most common mesenchymal tumors in the gastrointestinal tract, are increasingly treated with minimally invasive surgeries. Developed techniques include laparoscopic, endoscopic, and hybrid methods for gastric GIST resection. Our study, focusing on single-incision laparoscopic intragastric resection for gastric GISTs, aims to evaluate its safety, efficacy, and long-term outcomes.

**Materials and Methods:**

In a retrospective study of GIST surgery involving 14 patients who underwent single-incision laparoscopic intragastric resections, we analyzed and compared their preoperative demographics, American Society of Anesthesiologists (ASA) scores, tumor size, neoadjuvant treatment, operation duration, hospital stay, mitotic and Ki-67 indexes, and histological features with those of patients who underwent open and laparoscopic wedge resections, to assess the impact on both survival and disease-free survival.

**Results:**

Average operation time was 93.07 minutes (range 81-120 minutes). Average blood loss: 67 ± 20 mL (range 40-110 mL). Postoperative hospital stay averaged 6.79 days (range 4-16 days). Strong correlations were observed between preoperative and pathological tumor sizes (*P* = .001, *P* < .001). Survival analysis indicated a significant association with ASA scores (*P* = .031), but not with mitotic index, Ki-67, or tumor size. Average survival was 80.57 months, with no recurrence or metastasis during follow-up.

**Conclusion:**

Based on our experience, the single-incision laparoscopic intragastric resection method emerges as a highly efficient, time-saving, and gentle oncological procedure, providing a safe and minimally invasive alternative resulting in shorter hospital stays and excellent long-term outcomes with minimal recurrence. For more definitive conclusions, larger, multicenter, and prospective studies are recommended.

**Main Points**

- The single-incision laparoscopic intragastric resection is tailored based on tumor size and location, delivering superior outcomes and efficient specimen removal.
- A glove port boosts maneuverability and visibility, lowers contamination risk, and facilitates organ preservation and gastrostomy closure.
- Demonstrated feasibility and long-term oncological advantages indicate the technique’s potential for broader application, including treatment of early gastric cancers and other lesions.

## Introduction

Gastrointestinal stromal tumors (GISTs) are the most prevalent mesenchymal tumors in the gastrointestinal tract, with a preference for the stomach and small intestine. Approximately 50%-60% of GISTs originate in the stomach. Due to their potential malignancy, surgical removal, especially of larger GISTs, is often required.^1^ Open surgery has been the standard traditional approach but carries risks such as damage to surrounding tissues and extended recovery times.

In recent years, laparoscopic surgery, along with other minimally invasive techniques, has gained popularity. Laparoscopic surgery is less invasive than open surgery, leading to reduced patient discomfort, shorter hospital stays, quicker recovery, and lower risk of hospital-acquired infections. These benefits not only enhance patient outcomes but also offer greater cost-effectiveness. However, conventional laparoscopic approaches can be limited by the need for multiple incisions, challenges with manipulation, and associated risks of infection, herniation, and cosmetic issues. The selection of laparoscopic surgery for gastric submucosal tumors has been researched to depend on preoperative tumor location, improving accuracy in defining gastric resection extent and technical complexity.^2^

Laparoscopic intragastric resection excels in removing GISTs embedded in the gastric wall, offering improved gastric preservation and better access to the lesions.^3^ This method not only effectively treats gastric GISTs but also maintains a high standard of safety and feasibility, resulting in favorable oncologic outcomes. The technique initially evolved to incorporate multiple ports to increase manipulation capacity,^4^ and later progressed to using a single port for intragastric resection.^5-8^ Single-incision laparoscopic intragastric resection represents a more advanced evolution of this method, specifically designed for the management of gastric lesions. It offers notable advantages such as improved visualization, enhanced manipulation, and a smaller gastrotomy, thereby minimizing surgical impact.

This study conducts a retrospective analysis of the safety, efficacy, and complication rates associated with single-incision laparoscopic intragastric resection of GISTs. The primary aim is to evaluate the effectiveness of this minimally invasive procedure as a treatment option for gastric GISTs, while also examining the long-term outcomes for patients. Herein, we also report the comparison of outcomes between single-incision laparoscopic surgery, laparoscopic wedge resections, and open surgeries in our series.

### Materials and Methods

**Figure 1.**
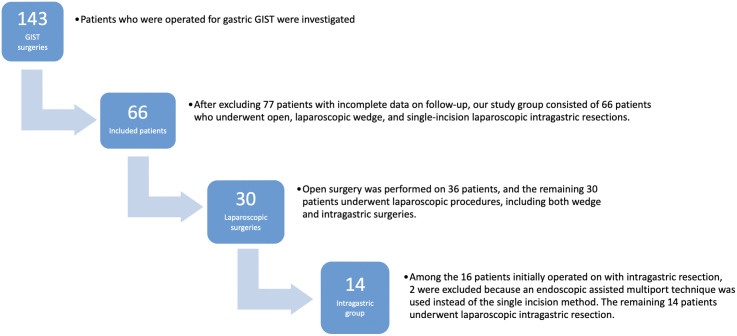
Flow diagram of the patient population. GIST, gastrointestinal stromal tumors.

Patients undergoing single-incision laparoscopic intragastric resection of gastric GISTs at the Department of General Surgery, Ege University, between 2012 and 2018, were retrospectively included in this study through an examination of the Electronic Patient File system. Of the initial cohort of 143 patients who underwent GIST surgery, the study ultimately included 66 patients after excluding cases with missing data or irregular follow-up. Patients who underwent open surgery, laparoscopic wedge resection, and single-incision laparoscopic intragastric resection were included in our group. This selection ensured the inclusion of only those patients with comprehensive data spanning the entire treatment and follow-up period (Figure 1).

### Surgical Technique

Patients were positioned supine in a split leg position with the surgeon between the patient’s legs and an assistant on the left side of the patient. A 3 cm midline supra-umbilical incision was made under general anesthesia, and the stomach was exteriorized through this incision using Babcock forceps. To facilitate traction of the anterior gastric wall, two 3-0 silk stay sutures were placed around the gastrostomy site before a 2 cm gastrostomy was created and opened with an electrocautery. A 2 cm incision was then made between the sutures, and the glove port circular cuff was inserted into the gastrotomy (Supplementary Video 1).

**Figure 2.**
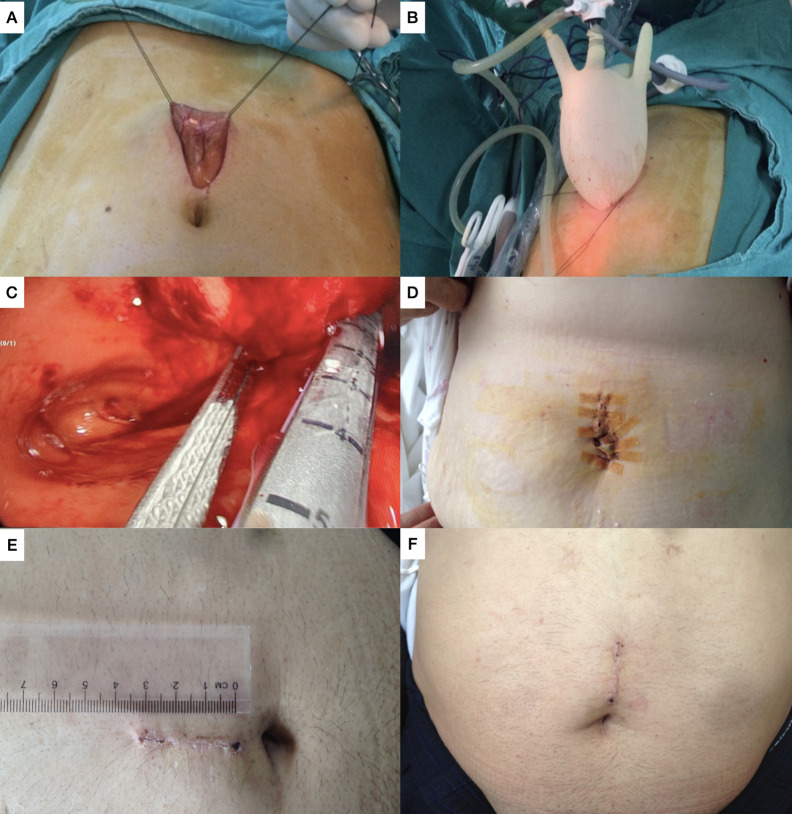
Steps of the surgery. A. Midline incision. B. Glove port C. Intragastric stapling D. Wound closure E. Length of incision. F. Postoperative third month.

A custom homemade glove port was prepared at the backtable and placed at the gastric entry, and the stomach was inflated with CO_2_ to a pressure of 10-12 mmHg for examination of the gastric mucosa using a 30° laparoscope (Figure 2). Inflation revealed that the 4 cm diameter cuff acted as a valve, securing the stomach in place without requiring any additional skin fixation. Hemostats were used to secure the stay sutures on either side of the gastrotomy, with both sutures hanged through the incision to provide additional support. Notably, no slippage or misplacement occurred after insufflation, possibly due to the pneumogastrium positioning the stomach closer to the skin, eliminating the need for further fixation.

Once the lesion was identified, one 10-mm and two 5-mm trocars were inserted through the index, middle, and ring fingers of the glove, each sutured or secured with rubber bands to form a disposable device.

Adjacent healthy tissue was sutured near the lesion for safe retraction and exposure. The lesion was then completely excised using 1 or 2 laparoscopic linear staplers. The resected specimen was removed in a specimen bag or through the glove port according to the size. The gastric cavity was cleaned, hemostasis achieved, and the suture line inspected, with endoclips or energy devices applied as needed for hemostasis. The 2 cm gastrotomy site was closed with a continuous interlocking full-thickness suture and reinforced with absorbable suture material for seromuscular closure. Since staplers were used for lesion resection following elevation into the lumen, suturing was not necessary for any of our patients. In our clinic, we usually do not suture the staple line or perform leak tests such as methylene blue during sleeve gastrectomy procedures. Therefore, we did not conduct any leak tests in this patient cohort, especially considering the intragastric nature of the resection. We do not conduct leak tests for gastric anastomoses as per our standard practice, and none were performed for gastrotomy site closure.

Drainage was applied selectively. After careful wound irrigation, the abdominal incision was sutured in layers. Postoperatively, patients received intravenous proton pump inhibitor twice daily for 3 days, then once daily. The nasogastric tube was removed on the first postoperative day, and the drain was usually removed on the third day, unless complications occurred. A liquid diet was started on the second postoperative day, followed by a soft diet.

### Statistical Analysis

Demographic data, American Society of Anesthesiologists (ASA) scores, tumor characteristics, which are determined during the preoperative and postoperative period, operation times, neoadjuvant and adjuvant treatment conditions, and survival time of 14 patients were collected in Excel format and transferred to SPSS version 26.0. The data were analyzed using the Statistical Package for the Social Sciences version 26.0 (IBM Corp., Armonk, NY, USA). Descriptive statistics were performed, and Kolmogrov–Smirnov was used to evaluate normality. For normally distributed data, relationships between groups were assessed using student’s *t*-test and analysis of variance, while Mann–Whitney *U* and Kruskall–Wallis tests were used for non-normally distributed data. Correlation analysis was conducted using Pearson’s correlation.

**Ethics Committee Approval**

The study was conducted in accordance with the Declaration of Helsinki and was approved by the Ethics Committee of Ege University (protocol code: 23-9.1T/40, date of approval: September 21, 2023). No informed consent was requested from patients since this is a retrospectively designed study.

## Results

In the specified years, 143 patients were operated on for GIST in our clinic. Due to the aim of long-term follow-up in our study, 77 patients with irregular follow-ups or missing data were excluded from the study. Among the 66 patients with regular follow-ups, open surgery was performed on 36 patients (54.5%), laparoscopic wedge resection on 16 patients (24.2%), and transgastric laparoscopic wedge resection on 14 patients (21.2%). When patients were divided into 3 groups: open surgery, laparoscopic, and laparoscopic intragastric, no significant differences were found between these 3 groups in terms of age, gender, mitotic index, disease-free period, and overall survival (*P* = .412, *P* = .054, *P* = .124, *P* = .243, *P* = .304). However, statistical differences were observed in operation time, hospital stay duration, and the day oral intake was started (*P* = .001, *P* < .001, *P* = .001).

Of the 14 patients undergoing single-incision laparoscopic intragastric resection for gastric GISTs, 6 (42%) were male and 8 (58%) were female. These demographic details are presented in Table 1.

Our study did not include any patients with an ASA score of 4-5 or who received neoadjuvant therapy. We compared tumor size as determined by preoperative radiological and endoscopic ultrasonography (USG) to the dimensions reported in pathology findings. Table 1 presents data on tumor localization, mitotic index, Ki-67 score, surgical margin, and the degree of tumor aggressiveness as outlined in the pathology reports.

A statistically significant correlation was found between the radiologically assessed preoperative tumor size and the dimensions documented in pathology reports (*P* = .001, Pearson score: 0.782). Additionally, a statistically significant correlation was observed when comparing tumor sizes measured by endoscopic USG with those in pathology reports (*P* < .001, Pearson Score: 0.931).

The average operative time was 93.07 minutes with a range of 81-120 minutes. The average blood loss was 60 ± 20 mL, ranging from 40-110 mL. Our clinic has implemented Enhanced Recovery After Surgery (ERAS) protocols, which typically result in patient discharge on the fourth postoperative day, barring any complications. The average postoperative hospital stay for this group was 6.79 days, with a range of 4-16 days. One patient was categorized as Clavien–Dindo class 3A, necessitating total parenteral nutrition and a delayed initiation of oral intake due to epigastric pain and nausea, accompanied by a surgical site infection, thereby prolonging the hospital stay. The operative and postoperative management details are compiled in Table 2. In all of our patients, the skin incision was 2-3 cm in length. All of our patients healed well without any scar formation, except for 1 patient who required open surgery (Figure 2).

Upon analyzing the association between conversion to open surgical technique and tumor size, no significant correlation was observed between the radiological size, endoscopic USG size, or size as per pathology reports and the conversion to open surgery (*P* = .286, *P* = .182, *P* = .286, respectively). Additionally, the examination of the relationship between tumor exposure and localization revealed no statistically significant correlation (*P* = .697).

Subsequent analysis did not yield any statistically significant findings (*P* = .549, *P* = .264, *P* = .154, *P* = .173, respectively) regarding the association between adjuvant therapy, tumor characteristics, and outcomes in terms of overall survival or disease-free survival. However, a significant correlation was identified between ASA scores and both overall survival and disease-free survival (*P* = .031, *P* = .033, respectively).

In comparing disease-free survival with Ki-67 and mitotic index as reported by the pathological findings, there was no statistically significant correlation between these variables (*P* = .135, Pearson score: −0.506; *P* = .054, Pearson score: −0.525, respectively). Similarly, there was no statistically significant association between overall survival and either the Ki-67 or mitotic indices (*P* = .147, Pearson score: −0.494; *P* = .235, Pearson score: −0.340, respectively). Three patients with intermediate- to high-risk GIST received adjuvant tyrosine kinase inhibitor therapy after surgery. Notably, none of these patients experienced a recurrence during the follow-up period.

## Discussion

Gastrointestinal stromal tumors are the most common mesenchymal tumors found in the gastrointestinal tract, often requiring surgical intervention, especially for larger tumors. The incidence of intragastric lesions has increased due to the increased screening of the upper gastrointestinal tract. Although most of these lesions are benign, complete removal is recommended for pathological examination to rule out potential malignancy. Minimally invasive techniques have gained acceptability due to the risks associated with traditional open surgery, such as damage to neighboring tissues, resection of more extensive required stomach tissue leading to a compromise of gastrointestinal functions, and lengthy recovery time. If the size or location of the lesion makes endoscopic resection impossible, the standard transperitoneal laparoscopic approach is the next least invasive option.

### Evolution of Intragastric Laparoscopic Surgery

Since the first successful intragastric laparoscopic procedure was described by Ohashi^3^ in 1995, the technique has evolved with contributions from technological advancements such as single-port access and novel surgical approaches. Early techniques involved endoscopy-assisted single-trocar access to the stomach. Later, successful modifications were developed incorporating endoscopy-assisted techniques, the use of one or multiple intragastric trocars, and additional standard abdominal trocars to improve manipulation and complete excision of intraluminal lesions.^9-12^

When selecting the appropriate surgical procedure, it is crucial to consider tumor size, type, and mitotic index, as well as patient-related factors. Laparoscopic intervention may present challenges, including failure to attain a complete resection margin, tumor rupture, and potential tumor seeding into the abdomen. For lesions located near the gastroesophageal junction, pylorus, lesser curve, fundus, and cardia, this technique was inadequate, and the maneuvering capacity was limited. This led to a search for better visibility and access. Lim^13^ conducted a study comparing open surgery in gastric GIST resections to laparoscopic intragastric resection in suitable patients. The results indicated similar outcomes from an oncologic perspective.

To enhance accessibility in difficult locations and ensure negative margins without tumor rupture, additional trocars and different laparoscopic approaches have been investigated. Liao et al^14^ discovered similar results in their study of 207 patients who underwent various laparoscopic procedures to remove gastric GISTs, including gastrectomies (total, subtotal, distal, gastric stump, or proximal), wedge resection, transgastric resection, and seromuscular dissection, as well as laparoscopic intragastric submucosal dissection. The study demonstrated that laparoscopic surgery is a viable and minimally invasive option for treating GISTs. The study emphasized the importance of individualizing treatment plans based on tumor location and size.

Boulanger-Gobeil et al^15^ reported on 8 cases of laparoscopic intragastric GIST surgery. They used 6 trocars, 3 for standard abdominal access, and an additional 3 cuffed trocars for intragastric access. Endoscopy was also used to assist in eliminating air from the stomach through the esophagus. The utilization of cuffed trocars allowed for the elevation of the stomach to the abdominal wall and prevented air leakage. According to the study, laparoscopic gastric GIST surgery resulted in better preservation of gastrointestinal functions and less work loss compared to the open technique. The findings were based on a comparison between the 2 techniques.

Each of these techniques has its own set of benefits and drawbacks. In a study conducted by Conrad et al^4^ on 11 patients, 4 laparoscopic intragastric surgery methods were evaluated: endoscopic assisted, intragastric stapling, cuffed multiports, and single-incision intragastric techniques. The authors found that the use of an endoscopic approach improved the safety and accuracy of port placement for intragastric procedures, allowing for precise targeting by adjusting the port position away from the midline. The intragastric stapling technique is a rapid alternative that reduces the risk of perforations associated with energy-based resections and eliminates the need for extensive sutures after full-thickness cuts. Cuffed ports secure the stomach and prevent dislocation. Single-incision laparoscopic intragastric surgery achieves stomach suspension from the anterior abdominal wall without the need for suturing multiple defects, as port closure can occur through the mini-laparotomy. However, it was stated that the single-incision laparoscopic technique was limited by the lack of triangulation, which restricts the range of motion.

The existing literature on intragastric multiport surgery focuses on treating lesions of various origins at the gastroesophageal junction, such as leiomyoma, GISTs, and T1a gastric cancer.^16,17^ However, multiport entries increase the risk of contamination and require more sites of gastrotomy closures. Staplers are utilized for the closure of gastrotomy sites, while clips are employed to control bleeding. Certain studies have demonstrated superior outcomes, including reduced bleeding, when continuous suturing is applied along the stapler line.^18^ Zhang et al^19^ investigated the excision of lesions near the esophagogastric junction in 13 patients using their modified single-incision laparoscopic intragastric surgery with a subxiphoid incision. During a mean follow-up of 14 ± 4 months, there were no tumor recurrences. The investigators concluded that single-incision laparoscopic intragastric resection is a safe alternative surgical method for resection of gastric stromal tumors located near the esophagogastric junction. Law et al^20^ recently reported successful outcomes in a significant series of 22 patients treated with single-incision transgastric resection for intraluminal gastric GISTs using a reduced-port technique. The median surgery time was 101 minutes, and there were no conversions to open surgery, 30-day mortality, or recurrence during follow-up. The authors concluded that the laparoscopic method was effective in providing surgical clearance, facilitating tumor extraction, and ensuring secure closure, while maintaining low morbidity.

### Our Study Group

In our group, we evaluated patients’ ASA scores, tumor size, and depth of invasion as determined by radiological and endoscopic ultrasonography (EUS) examination, as well as tumor localization, preoperatively to assess their suitability for the laparoscopic intragastric procedure.

In our general surgery clinic, in earlier cases before refining and modifying our technique, we performed 2 procedures using an endolaparoscopic approach, commencing with endoscopy followed by laparoscopy. Initially, we utilized endoscopy to inflate the stomach, facilitating optimal entry for laparoscopic ports. Despite achieving clear visualization, the proximity of the lesion to the cardia posed challenges. Although the trocars were directed toward the lesion, inadequate triangulation impeded efficient maneuvering. Additionally, despite positioning the stapler to ensure a necessary safety margin, adequate elevation of the lesion base was lacking, rendering laparoscopic completion unfeasible. However, both cases proved challenging to manipulate, and air leakage through the esophagus and gastric ports hindered the creation of a pneumogastrium and visualization of the gastric cavity, necessary for identifying a safe resection plane. These patients were part of the laparoscopic intragastric treatment group and were not included in our study population. Due to the limitations of the multiport endolaparoscopic technique, we chose to utilize the single-incision laparoscopic intragastric technique. To enhance maneuverability and establish pneumogastrium, we modified our technique to utilize the gloveport system. The fixed manufactured single ports do not offer the same range of motion and maneuverability as the hand gloves; therefore, we employed the homemade hand glove technique, which we believe provides superior range of motion compared to conventional single ports manufactured by the industry.

We employed a blue endostapler linear cartridge with a height of 3.5 mm, designed for tissue thickness ranging from 1.25 to 1.75 mm. The use of endostaplers in sleeve gastrectomy and gastrectomies is common practice and endorsed by both expert consensus and the manufacturer’s recommendation. Given its routine use in our practice and its alignment with tissue thickness, it was the chosen stapler for the procedure.

Previous studies have used a laparoscopic bowel clamp at the jejunum near the ligament of Treitz to prevent air passage into the gastrointestinal tract. However, more recent works have examined the omission of the clamp and found no significant leakage that would interfere with the procedure.^20^ Consistent with this approach, we abstained from employing any clamps, especially since our goal was to keep the entire laparoscopic procedure intragastric, without introducing extra trocars for intraperitoneal access, and we encountered no issues with bowel distension.

Patients who were deemed suitable underwent the laparoscopic intragastric resection procedure. Our operative time data is consistent with the literature on minimally invasive surgery, demonstrating that this technique is time efficient and simple, and thus may be preferable to laparoscopic methods. In 2008, Privette et al^2^ evaluated the duration of operation, bleeding loss, length of hospital stay, and mortality in patients who underwent minimally invasive surgery. They concluded that the minimally invasive technique is a safe and effective method when all factors are considered. Laparoscopic surgery was terminated in only 1 patient (7.1%), and we had to convert to an open technique due to the proximity of the tumor to the trocar entrance, which was not anticipated by preoperative imaging, making intragastric resection impossible.

The literature emphasizes the importance of endoscopic ultrasonography evaluation and R0 resection.^21^ It affirms that minimally invasive surgery can be safely and efficiently performed on suitable patients. In this series, there was no need for intraoperative endoscopic intervention, and no injuries were encountered during tumor dissection that could lead to seeding. Surgical margins were consistently negative in all patients, including both the laparoscopic and open surgical cohorts. Within the single-incision intragastric group, all patients also had negative surgical margins, with the closest margin being delineated at 1-2 mm. Importantly, no patient required re-operation and there were no cases of recurrence. With one of the longest follow-up periods recorded, our series demonstrates that the single-incision laparoscopic intragastric approach is an oncologically safe procedure.

Our complications were categorized according to the Clavien–Dindo system, initially delineated in 2004 and widely embraced across surgical disciplines for grading adverse events. Among our cohort, 12 patients fell into Clavien–Dindo grade 1, necessitating routine postoperative monitoring. Grade 1 entails deviations from the typical postoperative trajectory that do not demand pharmacological, surgical, endoscopic, or radiological interventions. Permissible treatments include antiemetics, antipyretics, analgesics, diuretics, electrolytes, and physiotherapy. This grade encompasses bedside management of wound infections as well. Since 12 patients reported mild pain and nausea and were administered analgesics and antiemetics, they were classified under grade 1 even though there were no significant complications, and their subsequent postoperative recovery was uneventful. Two patients experienced Clavien–Dindo grade 2 complications, necessitating antibiotic therapy. These cases involved incisional dehiscence and seroma formation in the skin and subcutaneous tissue. Plastic and reconstructive surgery consultants evaluated the wounds and opted for secondary wound healing with prophylactic antibiotics. One patient encountered a grade 3A complication, which is defined as requiring surgical, endoscopic, or radiological intervention. In this class, complications are significant yet manageable without the need for general anesthesia. In this instance, purulent drainage was observed at the incision site, prompting a computed tomography (CT) scan that revealed a collection beneath the incision. The collection was drained under local anesthesia, and supplementary intravenous antibiotics were initiated.

In laparoscopic intragastric resections, increasing tumor size, achieving R0 resection, and improving disease-free survival are crucial parameters. Surgical margins were consistently negative in all patients in our cohort, including both the laparoscopic and open surgical cohorts. Within the single-incision intragastric group, all patients also had negative surgical margins, with the closest margin being delineated at 1-2 mm. Importantly, no patient required re-operation and there were no cases of recurrence. Our study compared the tumor size reported in pathology with the radiologic and EUS tumor sizes, revealing a strong correlation between them. According to research, preoperative imaging is more valuable than other imaging techniques (magnetic resonance imaging–computed tomography) in determining invasion depth, with EUS measurements being the most valuable.^22-24^

Nagata et al^23^ emphasized in their study that patients can return to their social lives earlier and tolerate oral feeding better with intragastric resection. Our data is consistent with the existing literature, with our uneventful patients being discharged around the fourth day. In one of our patients with a complicated postoperative course, we encountered a surgical site infection and delayed initiation of oral diet due to concomitant nausea. We implemented ERAS protocols where possible, emphasizing early mobilization and limiting drains and catheters. Extended hospitalizations mainly involved patients needing postoperative diversion, hindering full ERAS adherence. The current patient cohort in the hospital includes 14 patients, with an average length of stay being 6.79 days. However, due to the limited number of patients, the case where a patient, who developed a surgical site infection and required intravenous antibiotics as recommended by infectious diseases, had a prolonged hospital stay of 16 days. When this patient is excluded from the group of 14, the average length of stay decreases to 6.08 (range 4-7) days. The average day to start oral administration shifts from 2.14 to 2.08 days. While the patient with the extended stay due to complications contributes nearly 1 day to the average hospital stay, there has not been a significant decrease in the average value regarding the start of oral administration. Our approach has remained as compliant as possible with ERAS protocols.

When divided into 3 groups—open surgery, laparoscopic, and laparoscopic intragastric resections—and investigated, our study showed that there were no significant disparities in terms of age, gender, mitotic index, disease-free interval, and overall survival among these groups. This consistency in findings could be linked to the early lesion detection enabled by the widespread use of endoscopy and timely interventions. However, we did find significant differences in the length of the operation, hospital stay, and the commencement of oral intake.

The survival analysis revealed a significant correlation only between survival and ASA scores. No significant correlation was found between tumor characteristics, adjuvant treatment, mitotic index, Ki-67 value, and survival or disease-free survival. Novitsky et al^25^ examined long-term survival rates and found a disease-free survival rate of 92%. The study found that tumor size, histological characteristics, and general health status of patients can improve survival rates. Mitotic activity and Ki67 proliferation index remain the gold standard for predicting GIST recurrence. Interestingly, a Ki67 cutoff of 13% demonstrated improved specificity and sensitivity compared to the traditional 10% threshold.^26^ While PDGFRβ proved unreliable for recurrence prediction, it could potentially aid in cKIT-negative GIST diagnosis. Our study, despite limitations due to sample size, suggests no significant association between mitotic index and Ki-67 scores for disease-free survival. Notably, 3 patients with intermediate-to-high-risk GISTs who received adjuvant tyrosine kinase inhibitor treatment following surgery remained recurrence-free throughout the follow-up period.

Gastric cancer poses a significant global health threat with high mortality rates. Early detection and resection are crucial for improving prognosis, making advancements in minimally invasive techniques like endoscopic submucosal dissection (ESD) particularly valuable. ESD enables en bloc resection for select early gastric cancers, potentially offering a cure. However, a rare scenario when GISTs appear concurrently with adenocarcinomas in the stomach should also be kept in mind.^27^ These distinct tumor types, originating from different cell layers, pose a diagnostic challenge due to their unclear coexistence. Endoscopic resection, potentially combined with endoscopic ablative therapies, can serve as an effective primary treatment for both GISTs and early gastric cancers.^28^ For patients unsuitable for ESD, endolaparoscopic hybrid methods offer a promising alternative, while intragastric resections provide a solution for endoscopically unmanageable early gastric cancer cases. Future studies are crucial to evaluate the efficacy of these approaches and optimize patient management in this unique clinical presentation.

Our study is limited by a small patient cohort and its retrospective, single-center design, challenges further amplified by the infrequency of single-incision intragastric surgery cases. An ideal study would perform a comparative analysis of the single-incision intragastric technique against established laparoscopic resection methods, such as conventional laparoscopic wedge or distal gastrectomy and multiport laparoscopic intragastric resections, which involve multiple gastrostomy sites. In our series, we managed to compare only the laparoscopic wedge resection and open surgery with the single-incision intragastric approach. While our findings add valuable insights to the limited existing literature on single-incision laparoscopic intragastric surgery, further research with a larger patient sample comparing all techniques is necessary to draw more comprehensive conclusions.

In conclusion, when selecting patients for laparoscopic intragastric resection of gastric GISTs, it is important to consider tumor size, localization, and ASA score. Based on our experience, the single-incision laparoscopic intragastric resection method emerges as a highly efficient, time-saving, and gentle oncological procedure, providing a safe and minimally invasive alternative. This method requires only 1 incision and surpasses the multiport technique in facilitating specimen extraction. Key benefits of this technique include preservation of organ integrity, enhanced maneuverability, improved visibility by eliminating air leakage, reduced risk of intra-abdominal contamination, and the ability to remove large specimens through a single incision. Additionally, this technique ensures a comprehensive closure of the gastrostomy site and mitigates complications associated with the use and closure of multiple trocars in the stomach’s anterior wall. The technique of homemade gloves enhances manipulation and prevents air leakage, making it effective for resecting low-aggressive tumors with clear margins.

Although this study is retrospective and single-center, expanding the patient cohort and conducting multicenter, prospective studies could yield more definitive outcomes.

*The video file linked to this article is available in the online version of the journal. Or you can utilize the QR code given on the Supplementary Video 1 to access the video.

## Figures and Tables

**Supplementary Video 1:**
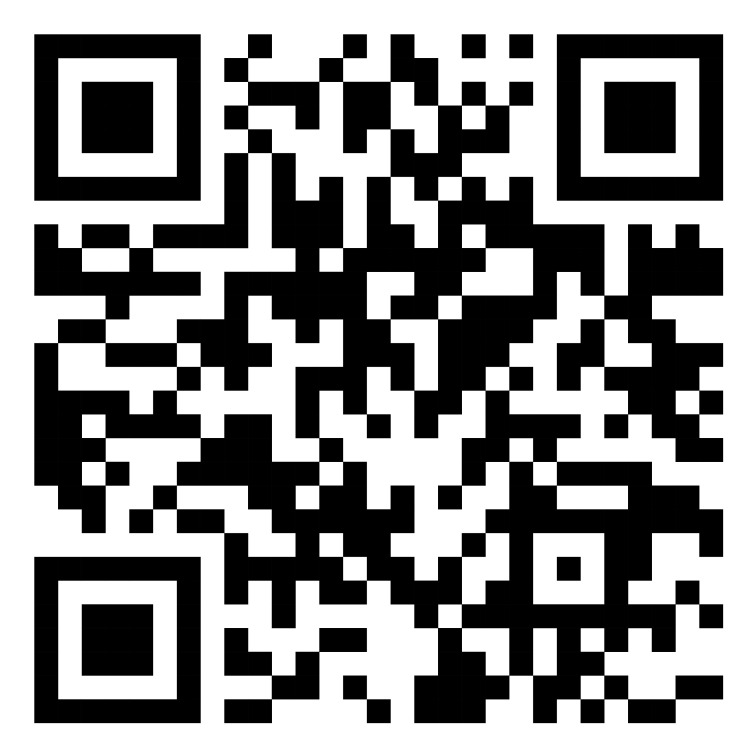
. Video of preparation of glove port and single incision laparoscopic intragastric surgery.

**Table 1.** Patient and Tumor Characteristics

Patient and Tumor Characteristics	Features	Intragastric (Median, Range, %)	Open (Median, Range, %)	Laparoscopic (Median, Range, %)	*P*
Age	3	61 (31-72)	63 (42-87)	70 (33-86)	.349
Gender	Male	6 (42.90%)	22 (61.10%)	4 (25%)	3
3	Female	8 (57.10%)	14 (38.90%)	12 (75%)	**.049**3
ASA Score	1	3 (21.40%)	30 (83.30%)	10 (62.50%)	3
3	2	8 (57.10%)	2 (5.60%)	6 (37.50%)	3
3	3	3 (21.40%)	4 (11.10%)	0 (0%)	3
3	4	0 (0%)	0 (0%)	0 (0%)	3
3	5	0 (0%)	0 (0%)	0 (0%)	**<.001**3
Radiologic tumor size	3	42 (15-105)	48 (5-300)	38 (20-60)	.423
EUS tumor size	3	32.09 (16-55)	67.29 (20-180)	32.80 (17-42)	.711
Reported tumor size	3	38.79 (3-80)	78.56 (8-240)	41.13 (17-80)	.579
Tumor location	Antrum	2 (14.30%)	12 (33.30%)	0 (0%)	3
3	Corpus	5 (35.70%)	18 (50.00%)	14 (87.50%)	3
3	Cardia	6 (42.90%)	4 (11.10%)	2 (12.50%)	3
3	Fundus	1 (7.10%)	2 (5.60%)	0 (0%)	**.008**3
Mitotic index	3	36% (20%-60%)	50% (10%-80%)	30% (20%-60%)	.15
Ki-67	3	6.62 (0-26)	5,77 (0-30)	7.75 (0-10)	.073
Tumor margin	Negative	14 (100%)	36 (100%)	14 (87.5%)	3
3	Positive	0 (0%)	0 (0%)	2 (12.5%)	**.04**3
Tumor behavior	Very low aggressive	10 (71.00%)	18 (50.00%)	10 (62.50%)	3
3	Low aggressive	2 (14.00%)	6 (16,70%)	6 (37.50%)	3
3	Medium aggressive	1 (7.00%)	4 (11.10%)	0 (0%)	3
3	High aggressive	1 (8.00%)	8 (22.20%)	0 (0%)	.128

Values in bold indicate statistical significance.EUS, endoscopic ultrasonography.

**Table 2.** Operative and Postoperative Information

Operative and Postoperative Information		Intragastric (Median, Range, %)		Open (Median, Range, %)		Laparoscopic (Median, Range, %)		*P*
Operation time	3	93.07	(81-120)	113.06	(90-140)	111.25	(90-130)	**<.001**3
Peroperation endoscopy need	3	0	0%	0	%0	0	0%	3
Conversion to open technique	3	1	7.10%	-	-	1	6.25%	3
Tumor rupture	3	0	0.00%	0	0.00%	0	0.00%	3
Oral intake opening	3	2.14	(1-3)	1.61	(1-3)	1.25	(1-2)	**<.001**3
Clavien–Dindo	1	12	85.70%	36	100%	16	100%	3
3	2	1	7.10%	0	0%	0	0%	3
3	3A	1	7.10%	0	0%	0	0%	.105
Hospital stay	3	6.79	(4-16)	11.78	(6-20)	8.63	(4-17)	**<.001**3
Adjuvant treatment	3	2	14.30%	10	27.80%	2	12.50%	.358
Survival	3	80.57	(39-121)	87.39	(73-102)	86.63	(72-103)	.321

Values in bold indicate statistical significance.
